# Providing an Additional Electron Sink by the Introduction of Cyanobacterial Flavodiirons Enhances Growth of *A. thaliana* Under Various Light Intensities

**DOI:** 10.3389/fpls.2020.00902

**Published:** 2020-06-25

**Authors:** Suresh Tula, Fahimeh Shahinnia, Michael Melzer, Twan Rutten, Rodrigo Gómez, Anabella F. Lodeyro, Nicolaus von Wirén, Néstor Carrillo, Mohammad R. Hajirezaei

**Affiliations:** ^1^Molecular Plant Nutrition, Department of Physiology and Cell Biology, Leibniz Institute of Plant Genetics and Crop Plant Research, Seeland, Germany; ^2^Instituto de Biología Molecular y Celular de Rosario (IBR-UNR/CONICET), Facultad de Ciencias Bioquímicas y Farmacéuticas, Universidad Nacional de Rosario, Rosario, Argentina

**Keywords:** *A. thaliana*, cyanobacteria, flavodiiron proteins, photosynthesis, electron sink, primary metabolism, biomass

## Abstract

The ability of plants to maintain photosynthesis in a dynamically changing environment is of central importance for their growth. As the photosynthetic machinery is a sensitive and early target of adverse environmental conditions as those typically found in the field, photosynthetic efficiency is not always optimal. Cyanobacteria, algae, mosses, liverworts and gymnosperms produce flavodiiron proteins (Flvs), a class of electron sinks not represented in angiosperms; these proteins act to mitigate the photoinhibition of photosystem I under high or fluctuating light. Here, genes specifying two cyanobacterial Flvs have been expressed in the chloroplasts of *Arabidopsis thaliana* in an attempt to improve plant growth. Co-expression of *Flv1* and *Flv3* enhanced the efficiency of light utilization, boosting the plant’s capacity to accumulate biomass as the growth light intensity was raised. The *Flv1*/*Flv3* transgenics displayed an increased production of ATP, an acceleration of carbohydrate metabolism and a more pronounced partitioning of sucrose into starch. The results suggest that Flvs are able to establish an efficient electron sink downstream of PSI, thereby ensuring efficient photosynthetic electron transport at moderate to high light intensities. The expression of Flvs thus acts to both protect photosynthesis and to control the ATP/NADPH ratio; together, their presence is beneficial for the plant’s growth potential.

## Introduction

Plant growth and development, fueled by photosynthesis, depend on the capture of light energy, a process carried out by the chloroplast (Stitt et al., 2010). Photosynthesis can be down-regulated by many factors, including an inadequate pool of ATP or an imbalance between the quantity of ATP and NADPH present (Avenson et al., 2005; Cruz et al., 2005; Amthor, 2010). Additional ATP is provided by the cyclic electron transport (CET) pathway, without production of extra NADPH. Photosynthesis is also affected by environmental conditions that might limit CO_2_ availability (e.g., stomatal closure) or CO_2_ assimilation by inhibition of the Calvin-Benson cycle (Czarnocka and Karpiński, 2018). One of the consequences of decreased photosynthetic efficiency is the over-reduction of the photosynthetic electron transport chain (PETC) and the stroma due to limitations in oxidized acceptors (NADP^+^), which may in turn promote the production of reactive oxygen species (ROS) by adventitious electron and/or energy transfer to O_2_ (Gómez et al., 2019). Since the functioning of both PSI and PSII are compromised by the presence of ROS (Miyake, 2010), the result is a further decrease in the plant’s capacity to assimilate CO_2_ (Zivcak et al., 2015a, b; Takagi et al., 2016). Alterations in plastid redox poise and ROS build-up affect chloroplast signaling and nuclear gene expression (Gollan et al., 2017). Thus, avoiding PSI electron acceptor limitation or introducing additional electron dissipating pathways into the chloroplast have the potential to improve photosynthetic efficiency and hence increase plant’s productivity.

Algae, cyanobacteria, non-vascular plants (mosses and liverworts) and gymnosperms have evolved an alternative electron flow (AEF) pathway, driven by the so-called flavodiiron proteins (Flvs). Analysis of the genome of the cyanobacterium *Synechocystis* sp. PCC6803 has identified the presence of four *Flv* genes. Their products, in the form of the heterodimers Flv1/Flv3 and Flv2/Flv4, drive oxygen-dependent electron flow under low (ambient) levels of CO_2_ availability and fluctuating and/or high light conditions (Zhang et al., 2012; Allahverdiyeva et al., 2013; Hayashi et al., 2014; Shimakawa et al., 2015). The Flv1/Flv3 heterodimer generates an electron sink downstream of PSI and directs the electron flow to reduce O_2_ to H_2_O without ROS formation, thereby protecting PSI (Allahverdiyeva et al., 2013). According to Yamamoto et al. (2016), expression of the *Physcomitrella patens* Flv1/Flv3 orthologs in the *Arabidopsis thaliana pgr5* mutant (deficient in the main CET pathway) provides partial compensation for the impairment of CET, demonstrating that Flvs could be functionally beneficial in an angiosperm. Shimakawa et al. (2017) have shown that in the liverwort *Marchantia polymorpha*, Flv1/Flv3 contributes to P700 oxidation and hence protects PSI against photoinhibition. When Gómez et al. (2018) co-expressed *Synechocystis Flv1* and *Flv3* in tobacco chloroplasts, the photosynthetic performance of the resulting transgenic plants under steady-state illumination proved to be comparable to that of wild-type (WT) leaves, while the induction of electron transport and non-photochemical quenching during a dark-to-light transition was significantly faster. Also, expression of *P. patens Flv1* and *Flv3* in the rice *pgr5* or *NDH* mutants has been shown to rescue biomass accumulation (Wada et al., 2018). Moreover, the enhancement of ATP synthesis resulting from over-expression of *Flv3* in *Synechocystis* led to accumulation of glycogen and a consequent increase in cell dry weight (Hasunuma et al., 2014).

The purpose of the present investigation was to determine the phenotypic effect of expressing *Synechocystis Flv1/Flv3* genes in *A. thaliana* plants grown under various light intensities. The focus was to establish whether heterologously expressed Flvs could act as electron sink within the PETC, and if so, whether this capacity had the potential to boost the plant’s productivity.

## Materials and Methods

### Expression of Cyanobacterial Flv1/Flv3 in *A. thaliana* and Localization of the Transgenic Products

**FIGURE 1:**
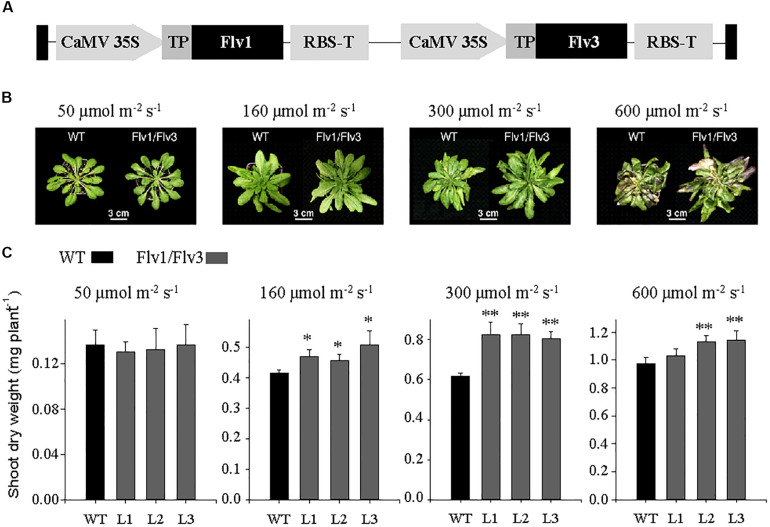
Growth of *A. thaliana* plants heterologously expressing cyanobacterial *Flv* genes. **(A)** Design of the transgene construct pCHF3-*Flv1/Flv3*. The sequence encoding the transit peptide of pea FNR (TP, gray box) was fused to the 5′-end of each *Flv* coding region to target the corresponding fusion products to chloroplasts. Each construct was placed between individual CaMV 35 promoters and RBS (RubiscoS-E9) terminators. **(B)** Phenotypes of 6 weeks old plants exposed to an 8-h photoperiod at a variety of light intensities. **(C)** Shoot dry weight: data are shown as means ± SE (*n* = 5–8). *, **: means differ from the performance of WT at *P* ≤ 0.05 and *P* ≤ 0.01, respectively. L1–L3 represent three independent stable lines expressing Flv1/Flv3 in chloroplasts.

*Arabidopsis thaliana* Col-0 lines constitutively expressing *Flv1* and *Flv3* were generated by floral dip (Clough and Bent, 1998), using the pCHF3-derived plasmid described by Gómez et al. (2018), in which the two *Flv* genes were cloned in the same vector backbone between the left and right borders of T-DNA (Figure 1A). Transgene expression was driven by separate cauliflower mosaic virus (CaMV) 35S promoters, and a sequence encoding a pea ferredoxin-NADP^+^ reductase (FNR) transit peptide was fused in-frame to the 5′-end of each *Flv* gene to direct expression of the corresponding products to the chloroplast (Figure 1B). Homozygotes were selected by segregation analysis and confirmed by proportional increases in gene contents as determined by PCR amplification with the primers given in Supplementary Table S1 (*Flv1* F/R and *Flv3* F/R). Three independent transformants were developed into stable transgenic lines (L1-L3). *Flv* transcript levels were monitored using a quantitative real-time PCR (qRT-PCR) assay (Supplementary Figure S1).

Chloroplast targeting of the transgenes was validated by fusing the *GFP* sequence (encoding green fluorescent protein) to the 3′-end of the *Flv* coding regions, taking advantage of PGBW5 Gateway binary vectors driven by the CaMV 35S promoter (Supplementary Figure S2a). Vectors containing the *Flv* transgenes were transferred into *Agrobacterium tumefaciens* strain EHA105 by the Dower et al. (1988) electroporation method and thence into leaves of *Nicotiana benthamiana* using agroinfiltration as described by Sainsbury and Lomonossoff (2008). Leaves sampled 48 h after infiltration were subjected to confocal laser scanning microscopy (CLSM) to monitor GFP fluorescence (Supplementary Figure S2b).

### Growth Conditions

**FIGURE 2:**
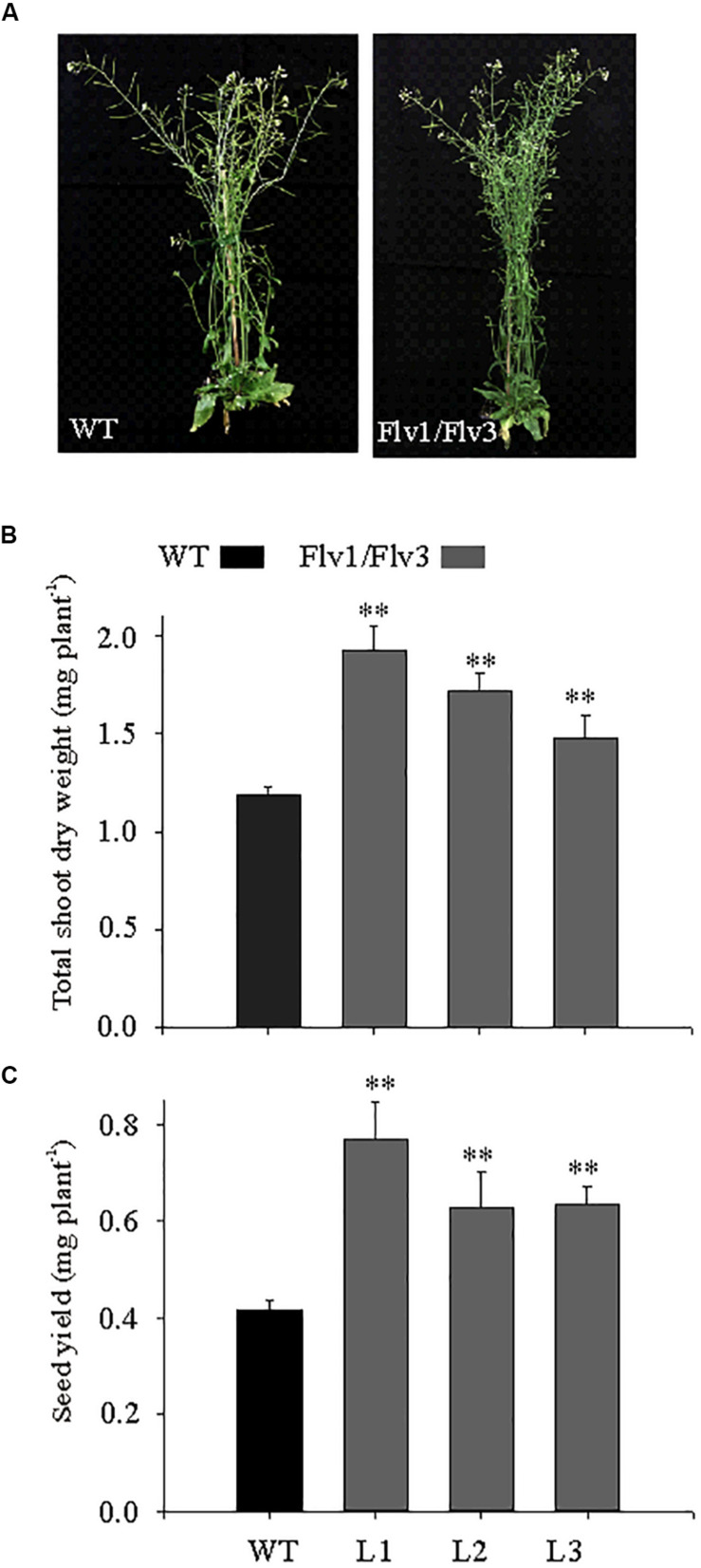
The effect of heterologously expressing *Flv1/Flv3* genes on the development of *A. thaliana* plants grown under long-day photoperiod. **(A)** Phenotypes of WT and *Flv* transgenics. Plants were grown for 6 weeks under a 16-h photoperiod (160 μmol photons m^–2^ s^–1^ of actinic light) and then transferred to ambient conditions for another 3 weeks for determination of **(B)** total shoot dry weight and **(C)** seed yield per plant. Data shown as means ± SE (*n* = 8). **: means differ from the performance of WT plants at *P* ≤ 0.01.

Seeds of WT and *Flv*-expressing lines were surface-sterilized by immersion in 70% (v/v) ethanol and 0.05% (v/v) Tween-20 for 15 min, and then rinsed in 96% (v/v) ethanol for 30 s. After air drying, seeds were held at 4°C for 48 h, plated on vertically oriented agar containing half strength Murashige and Skoog (1962) medium and grown under an 8-h photoperiod (160 μmol photons m^–2^ s^–1^) at 22°C. After 2 weeks, seedlings were potted into a mixture of 70 L substrate 1 (Düsseldorf, Germany), 23 L vermiculite and 372 g plantacote depot 4 m, and held at 22°C, 80% relative humidity under an 8-h photoperiod provided by Master HPI-T Plus 250 W fluorescent lights (Philips, Netherlands) at four different light intensities: low (50 μmol photons m^–2^ s^–1^), moderate (160 μmol photons m^–2^ s^–1^), moderately high (300 μmol photons m^–2^ s^–1^) and high (600 μmol photons m^–2^ s^–1^). The CO_2_ level was maintained at 400 ppm and plants were kept fully hydrated. For determination of shoot dry weight, whole rosettes of individual plants were harvested at the end of 6 weeks from date of germination. The plant material was dried for 16 h at 80°C and individual rosette dry weights were measured. For leaf biochemical analyses, rosettes of 6 weeks old plants exposed to 160 μmol photons m^–2^ s^–1^ and harvested at various time points during the diurnal cycle (0, 4, 8, 16, 20, and 24 h) were snap-frozen in liquid nitrogen and ground to powder. Plants were also grown under long-day conditions (16-h photoperiod, 160 μmol photons m^–2^ s^–1^) for 6 weeks, with all other environmental parameters identical to those used for the short-day grown plants. For determination of total shoot dry weight and seed yield, plants were transferred to the growth conditions with 12 h photoperiod and 160 μmol photons m^–2^ s^–1^ for another 3 weeks (Figure 2).

### Determination of the Leaf Contents of Carbohydrates, Amino Acids and Metabolites

For the determination of soluble sugars (glucose, fructose, and sucrose) and amino acids, a 50-mg aliquot of powdered frozen leaf tissue was extracted in 0.7 mL of 80% (v/v) ethanol at 80°C for 1 h. Following centrifugation (18,700 *g*, 10 min), the supernatant was evaporated under vacuum at 40°C, and the residue dissolved in 0.2 mL deionized water. Sugar contents were quantified using the enzymatic method of Ahkami et al. (2013), while those of the individual amino acids were determined according to Mayta et al. (2018). The pelleted material was used to assess the leaf’s starch content: pellets were rinsed twice in 80% (v/v) ethanol, air-dried at 80°C for 1 h and resuspended in 0.2 M KOH. The resulting suspension was held at 80°C for 1 h, the pH adjusted to neutrality using 1 M acetic acid, then incubated overnight at 37°C in 50 mM NaAc (pH 5.2) containing 7 units mg^–1^ amyloglucosidase. The glucose thereby released was measured as above. The methods used for the quantification of primary metabolites followed Ghaffari et al. (2016).

### Determination of the Leaf Contents of Adenine Phosphates

Adenine phosphates were quantified employing an UPLC-based method developed from that described by Haink and Deussen (2003). Prior to the UPLC separation step, 20-μL aliquots of the samples used for the quantification of metabolites (as well as a mixture of ATP, ADP, AMP, and ADPGlc) were derivatized by the addition of 45 μL of 10% (v/v) chloracetaldehyde and 435 μL of 62 mM sodium citrate/76 mM KH_2_PO_4_ (pH 5.2), followed by a 40-min incubation at 80°C, cooling on ice, and centrifugation (20,000 *g*, 1 min). Reverse-phase UPLC separations were achieved using an Infinity 1200 device (Agilent, Waldbronn, Germany). The gradient was established employing eluents A (TBAS/KH_2_PO_4_: 5.7 mM tetrabutylammonium bisulfate/30.5 mM KH_2_PO_4_, pH 5.8) and B (a 2:1 mixture of acetonitrile and TBAS/KH_2_PO_4_); the Roti C Solv HPLC reagents were from Roth (Karlsruhe, Germany). The 1.8 μm, 2.1 × 50 mm separation column was an Eclipse plus C18. The column was pre-equilibrated for at least 30 min in a 9:1 mixture of eluents A and B. During the first 2 min of the run, the column contained 9:1 A:B, changed thereafter to 2:3 A:B for 2.3 min followed by a change to 1:9 A:B for 3.1 min and set to initial values of 1:9 for 2.6 min. The flow rate was 0.6 mL min^–1^ and the column temperature was maintained at 37°C. Excitation and emission wavelengths were 280 nm and 410 nm, respectively. Chromatograms were integrated using the MassHunter (release B.04.00) software (Agilent).

### Determination of the Leaf Content of Glutathione

Glutathione was extracted from leaves according to Davey et al. (2003). Approximately 100 mg of fresh leaf material were ground to fine powder using tissue homogenizer with 1 mM EDTA and 0.1% (v/v) formic acid at 4°C under green safe light and centrifuged at maximum speed (35,280 *g*) for 10 min. Measurements of oxidized and reduced glutathione were carried out immediately in freshly prepared extracts. Separation and analysis of the desired compounds were performed on a C18 column (HSS T3, 1.8 μm, 2.1 × 150 mm, Waters, Germany) and an UPLC/MS-MS (Infinity ll, 6490 Triple Quadrupole LC/MS, Agilent), respectively. Two μL of extracts and the corresponding standards were injected in the mobile phase consisting of purest water plus 0.1% (v/v) formic acid and pure methanol plus 0.1% (v/v) formic acid. The temperatures of the auto sampler and column were maintained at 8 and 37°C, respectively. Separated compounds were eluted at a flow rate of 0.5 mL min^–1^, and their quantification was performed using the MassHunter (release B.04.00) software.

### Transmission Electron and Confocal Laser Scanning Microscopy

Transmission electron microscopy was performed following Mayta et al. (2018). For ultrastructure analysis, 2-mm^2^ cuttings from the central part of three leaves from five different plants of WT and *Flv1/Flv3*-harboring lines were used for conventional and microwave-assisted fixation substitution and resin embedding as detailed in Supplementary Table S2. Sectioning and electron microscopy analysis were performed as described previously (Kraner et al., 2017).

To estimate starch accumulation, 100 randomly selected chloroplasts of each WT and *Flv1/Flv3*-harboring plants have been used. Measurements of length, width, area and size of chloroplasts and starch granules were carried out with Image J software^1^. Furthermore, starch bodies per chloroplast have been counted.

Localization analysis of Flv1 and Flv3 fused to GFP at their C-termini and expressed in *N. benthamiana* cells was carried out by CSLM using a Zeiss LSM 780 microscope (Carl Zeiss GmbH, Jena, Germany). GFP was excited with a 488 nm laser line and fluorescence emission detected with a 491–535 nm band-pass filter.

### RNA Isolation, cDNA Synthesis and Transcription Analysis

Total RNA was extracted from young leaves following the protocol of Logemann et al. (1987), subjected to DNase treatment (Life Technologies, Darmstadt, Germany) and converted to ss cDNA using a RevertAid first strand cDNA synthesis kit (Life Technologies, Darmstadt, Germany) supplemented with a template of 1 μg total RNA and oligo dT primers. The reaction was carried out at 42°C for 60 min. The primers used for qRT-PCR analysis of *Flv* transgenes are listed in Supplementary Table S1 (*Flv1*-RT F/R and *Flv3*-RT F/R). The assays were performed in a CFX384 touch real-time system using the SYBR Green Master Mix Kit (Bio-Rad, Feldkirchen, Germany). The relative expression for *Flv* genes was calculated based on the expression of the house-keeping gene *Ubi10* (GenBank accession number *At4g05320*), as WT plants did not contain *Flv* genes. Primers employed to amplify *Ubi10* are also given in Supplementary Table S1. Relative transcript abundances were determined with the ΔΔCt method according to Schmittgen and Livak (2008).

### Statistical Analysis

Means and standard errors (SE) were calculated using SigmaPlot software^2^. The Student’s *t*-test was employed to evaluate for the statistical significance of differences between means.

## Results

### The Growth Response of *Flv*-Expressing *A. thaliana* Plants to Variations in the Light Intensity

To generate *A. thaliana* plants expressing plastid-targeted Flv1 and Flv3, the coding regions of the corresponding *Flv* genes were fused in-frame to the 3′-end of a DNA sequence encoding the chloroplast transit peptide of pea FNR and placed under the control of the constitutive CaMV 35S promoter in plasmid pCHF3-*Flv1/Flv3* (Gómez et al., 2018; Figure 1A). Expression of the *Flv1/Flv3* genes was monitored by measuring the corresponding transcripts using qRT-PCR (Supplementary Figure S1). Chloroplast localization of the Flv products was confirmed by introducing a C-terminal GFP tag to both proteins (Supplementary Figure S2a) and transiently expressing them in *N. benthamiana*. Supplementary Figure S2b shows that GFP fluorescence was confined to plastids in both cases. Image analysis suggests that Flv3 was translocated to all chloroplasts, whilst Flv1 was only detected in a fraction of them (Supplementary Figure S2b), most likely affecting the effectivity of heterodimer formation in the transgenic plants. The levels of heterodimer accumulated in *Flv1/Flv3* cells were however sufficient to elicit a growth phenotype in the transformants (see below).

Homozygous lines L1–L3, belonging to the T3 generation, were used for phenotypic characterization. The development of biomass in both WT and *Flv* transgenic plants grown at various light intensities is illustrated in Figure 1B. When illuminated at 50 μmol photons m^–2^ s^–1^, the performance of the transgenic plants was not distinguishable from that of their WT counterparts. However, when the light intensity was increased to either 160 or 300 μmol photons m^–2^ s^–1^, the transgenic plants were clearly larger (Figure 1B). Comparisons of shoot dry weight indicated that transgenic plants harboring *Flv1*/*Flv3* out-performed WT siblings by 10–30% (Figure 1C). Plants expressing *Flv1*/*Flv3* also grew better at 600 μmol photons m^–2^ s^–1^ (Figure 1C), even though they looked stressed at this irradiation levels, as suggested by the color of the leaves, presumably due to anthocyanin accumulation as a typical response to high light (Figure 1B).

### The Effect of Expressing *Flv1/Flv3* Transgenes on Biomass Accumulation in Plants Grown Under Long-Day Photoperiod

Under a long-day regime, the *Flv* transgenics flowered earlier than WT plants (data not shown), and were more bushy, with increased inflorescences (Figure 2A). Shoot dry weight was up to 1.8-fold greater in *Flv*-expressing plants than in WT counterparts (Figure 2B). Seed size was unaffected by the presence of the transgenes (data not shown), but seed yield was 1.5- to 1.8-fold greater (Figure 2C).

### The Effect of Expressing *Flv* Transgenes on Leaf Sugar, Starch and Amino Acid Contents

Yamamoto et al. (2016) and Gómez et al. (2018) have reported that under steady-state illumination conditions, the photosynthetic activity of *Flv*-expressing plants did not differ significantly from their WT siblings. To determine if Flv1/Flv3 expression affected other central metabolic routes, the leaf contents of carbohydrates and amino acids were measured in plants grown at 160 μmol photons m^–2^ s^–1^, a condition that exhibited significant biomass gains in the transformants relative to the wild-type (Figure 1).

**FIGURE 3:**
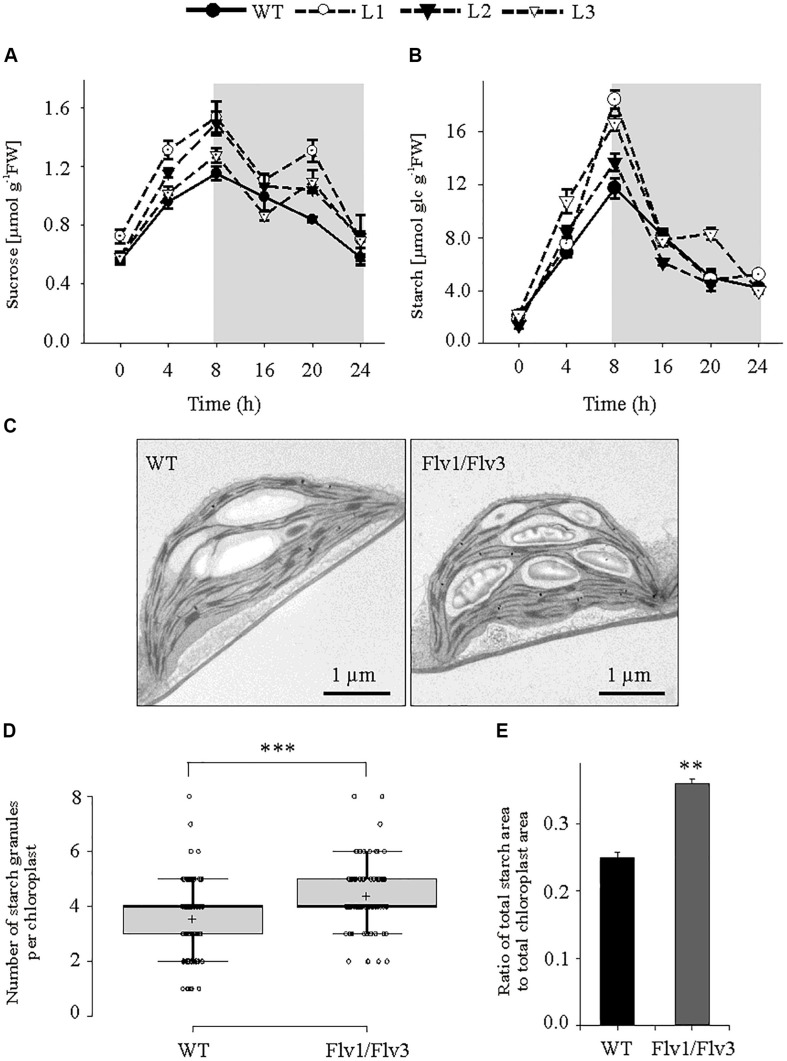
The effect of heterologously expressing *Flv1/Flv3* genes on diurnal variation in carbohydrate metabolism. Temporal variation in the rosette leaves of 6 weeks old plants exposed to 160 μmol photons m^–2^ s^–1^ of actinic light with respect to the contents of **(A)** sucrose and **(B)** starch. Gray boxes indicate the dark period. Data shown as means ± SE (*n* = 5). FW, fresh weight. **(C)** Representative images of starch granules present in leaves harvested after 5 h of light exposure. **(D)** Number of starch granules per chloroplast [*n* (chloroplasts) = 100]. **(E)** Ratio of total area of starch granules divided by the total area of chloroplasts. **,***: means differ from the performance of WT plants at *P* ≤ 0.01 and *P* ≤ 0.001, respectively.

Under this light regime, leaves of plants harboring *Flv1*/*Flv3* accumulated significantly higher sucrose concentrations than those of WT counterparts; in each line, sucrose contents increased gradually during the light period and fell during the dark period (Figure 3A). Leaf starch contents did not vary between genotypes at the beginning of the light period, but they increased faster (by as much as 1.7-fold) during the day in *Flv*-transgenic plants than in WT siblings (Figure 3B). Moreover, ultrastructural data obtained by transmission electron microscopy indicate that while the average size of starch granules was unaffected by *Flv* expression, their number was enhanced in the leaves of plants expressing *Flv1/Flv3* (Figure 3C). Counting starch granules of 100 individual chloroplasts from WT and Flv1/Flv3-expressing plants revealed that the total number of starch granules was 1.24-fold higher in Flv1/Flv3-expressing plants (440) compared to the WT (354) (Figure 3D). Furthermore, the ratio of the measured total area of starch granules divided by the total area of chloroplasts was higher by a factor of 1.44 in *Flv1/Flv3* expressing plants (680 μm^2^/1873 μm^2^) than in WT siblings (487 μm^2^/1912 μm^2^) (Figure 3E), indicating that the chloroplasts of *Flv1/Flv3* transgenic plants contained a higher starch volume.

Glucose and fructose contents failed to show consistent differences between lines during the entire photoperiod (Supplementary Figure S3). Also, no clear differences were observed with respect to the amounts of any of the amino acids following the plants’ exposure to 4 h of light (Supplementary Table S3), but by the end of the light period (8 h of light), an increased pool of asparagine, aspartate, glutamine and alanine was observed in *Flv1*/*Flv3* transgenics with respect to WT plants (Supplementary Table S4).

### *Flv1/Flv3* Expression Increased the ATP Levels of *A. thaliana* Leaves

**FIGURE 4:**
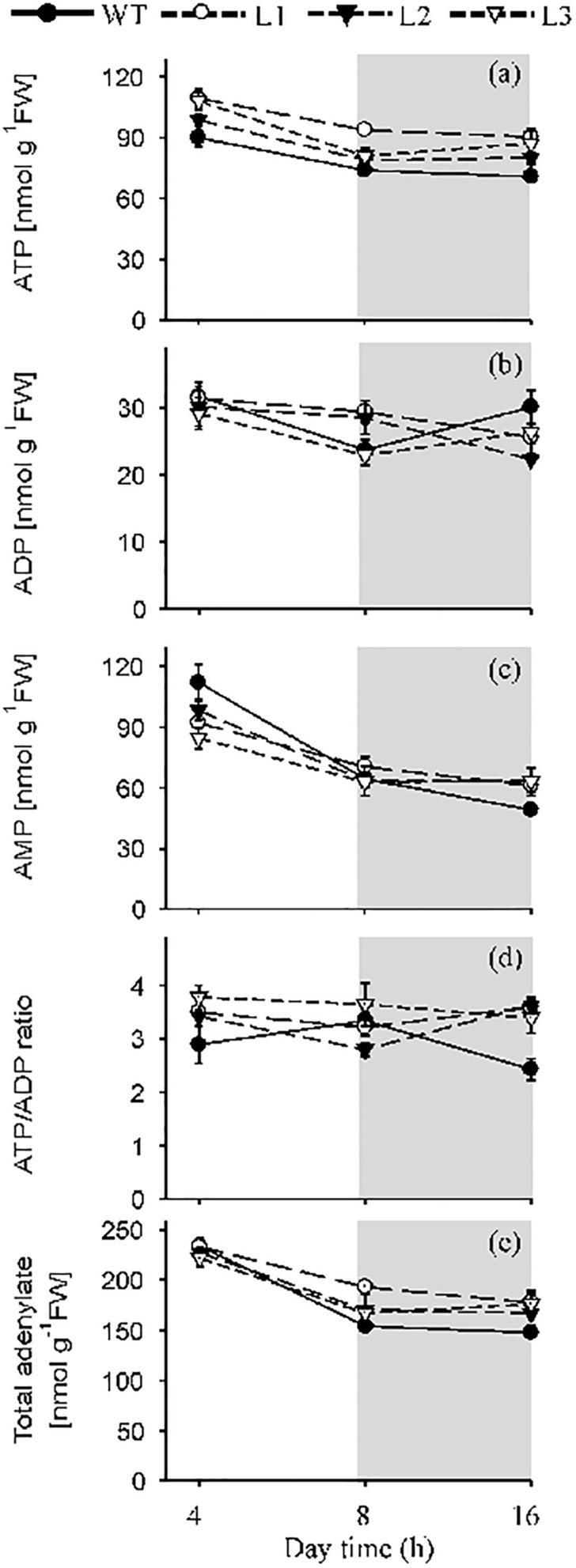
The effect of heterologously expressing *Flv1/Flv3* genes on the contents of ATP, ADP, AMP, the ATP/ADP ratio and total adenylates. Temporal variation in the rosette leaves of 6 weeks old plants exposed to 160 μmol photons m^–2^ s^–1^ of actinic light with respect to **(a)** the contents of ATP, **(b)** ADP and **(c)** AMP, **(d)** the ATP/ADP ratio, **(e)** total adenylates. L1–L3: independent lines harboring *Flv1/Flv3*. Data are shown as means ± SE (*n* = 5).

Leaf contents of ATP, ADP and AMP were measured during both the light (after exposure to 4 and 8 h of illumination) and dark periods (16 h). ATP contents were up to 1.25-fold (after 4 h of illumination), 1.3-fold (after 8 h of illumination only in L1) and 1.3-fold (after 8 h in the dark) higher in *Flv1/Flv3* leaves than in WT counterparts (Figure 4a). The leaf contents of ADP did not differ significantly between lines, whereas the AMP levels were significantly lower up to 1.3-fold in *Flv1/Flv3* transgenic plants compared to WT siblings (Figures 4b,c). The ATP/ADP ratio was thus higher at 4 and 16 h by a factor of 1.3- and 1.5-fold, respectively, in all the transgenic lines compared to that of WT siblings (Figure 4d). The total adenylate content failed to show differences between lines except for L1, where it was ∼1.2-fold higher than that of WT plants after 8 h of light (Figure 4e).

### The Effect of Expressing *Flv* Transgenes on the Contents of Glutathione

The contents of both the reduced and oxidized forms of glutathione (GSH and GSSG, respectively) were measured and their ratio was calculated. There was a significant decline of GSH in L3 plants whereas GSSG was statistically higher in line L2 of the transgenic leaves (Supplementary Figures S4a,b), resulting in a reduction in the GSH/GSSG ratio of up to 2.5 fold (Supplementary Figure S4c).

### The Effect of Expressing *Flv* Transgenes on the Leaf Metabolome

**FIGURE 5:**
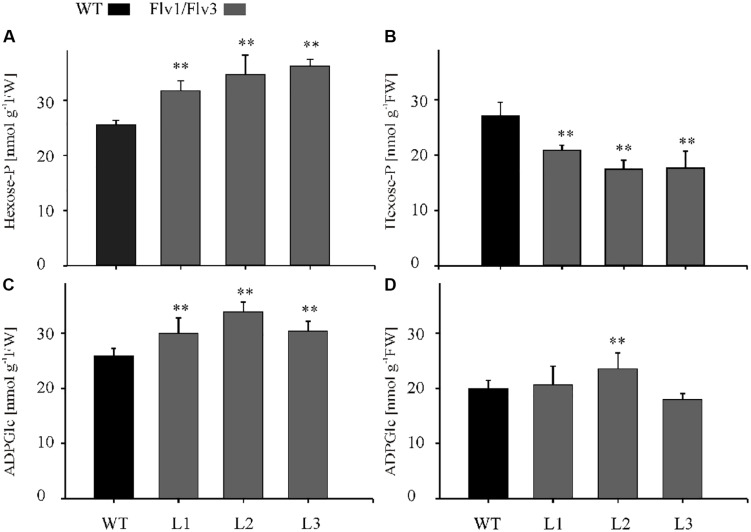
The effect of heterologously expressing *Flv1/Flv3* genes on the contents of primary metabolites. Hexose phosphates **(A,B)** and ADPGlc **(C,D)** were measured in the rosette leaves of 6 weeks old plants after exposure to 4 h **(A,C)** or 8 h **(B,D)** of light. L1–L3: independent lines harboring *Flv1/Flv3*. Data shown as means ± SE (*n* = 5). **: means differ from the performance of WT plants at *P* ≤ 0.01.

The contents of a number of metabolites were affected by the expression of *Flv* transgenes. After a 4-h exposure to light, the concentration of hexose phosphates was raised in all transgenic lines to a level significantly higher than that obtained in WT leaves (Figure 5A); however, by the end of the light period, hexose phosphate contents were significantly below those of WT leaves in all transgenic plants (Figure 5B). The concentration of the starch precursor ADPGlc was also elevated up to 1.4-fold above the WT level in the *Flv1/Flv3* transformants after 4 h of illumination (Figure 5C). When measured again after an 8-h exposure to light, the levels of ADPGlc remained statistically unchanged in the transgenics relative to WT siblings, with the exception of plants from line L2 (Figure 5D). With respect to malate, significant increases were only recorded in leaves of line L2 assayed after a 4-h exposure to light, whereas in those of line L3 malate levels were statistically lower after an 8-h exposure to light (Supplementary Figures S5a,c). A modest increase in the concentration of citrate was noted with respect to WT levels in the leaves of L3 plants at 4 h and L1 plants at 8 h of illumination (Supplementary Figures S5b,d).

## Discussion

Photosynthesis is essential for the growth and development of plants, but the process is relatively inefficient since just 8–10% of the overall spectrum of solar radiation is used to convert CO_2_ to sugar, while only 2–4% of incident light energy is channeled into growth (Long et al., 2006; Zhu et al., 2010). Most of the solar light intercepted by a leaf is lost by reflection, transmission and absorption by non-photosynthetic pigments, or is simply outside photosynthetically useful wavelengths. In C3 plants, less than 45% of the incident light is harvested, and still a substantial amount is released as heat and fluorescence, or used for photorespiration (Long et al., 2006; Zhu et al., 2010). In addition, suboptimal conditions such as water and CO_2_ limitation, high light, extreme temperatures, etc., might lead to over-reduction of the PETC and uncontrolled ROS production (Gómez et al., 2019). Avoiding these effects by establishing alternative electron sinks in chloroplasts can potentially enhance photosynthesis and overall plant growth.

Flvs have been reported to contribute to photosynthetic redox balance in a number of phototrophs (Jokel et al., 2018; Santana-Sánchez et al., 2019). Moreover, Flv1/Flv3 expressed in angiosperms were shown to act as electron sink for the PETC under certain circumstances (Yamamoto et al., 2016; Wada et al., 2018), especially during dark-light transitions (Gómez et al., 2018). However, the effect of this intervention upon growth and metabolism of the host plants was not reported in those articles. The aim of the present research was to evaluate this by co-expressing *Flv1* and *Flv3* in *A. thaliana* and monitoring growth and metabolic status in the corresponding transformants. The results indicate that plants harboring *Flv1/Flv3* grew significantly better under a range of moderate to high light intensities (Figure 1), and displayed increased carbohydrate and ATP levels (Figures 3, 4). The implication of these observations is that Flvs may act as regulators of photosynthesis when expressed in angiosperms, specifically avoiding over-reduction of the PETC as the electron pressure mounted up under increasing light intensities.

In cyanobacteria, the Flv1/Flv3 dimer provides an alternative electron sink at the acceptor side of PSI, preventing over-reduction of the PETC under adverse environmental conditions (Allahverdiyeva et al., 2013; Gerotto et al., 2016; Santana-Sánchez et al., 2019). Likewise, Gómez et al. (2018) have shown that tobacco plants expressing cyanobacterial *Flv1* and *Flv3* showed an improved ability of their dark-adapted leaves to maintain the PETC in a more oxidized state and to enhance proton motive force, again indicating a stronger electron sink in the transformants. The present results suggest a similar interaction of the introduced Flvs with the PETC in the transgenic *A. thaliana*. This hypothesis also agrees with the electron sink activity provided by Flvs in mutants deficient in CET under both high and fluctuating light (Yamamoto et al., 2016; Wada et al., 2018).

In phototrophic organisms, AEF pathways are induced shortly after exposure to light, contributing additional ATP to supply the Calvin–Benson cycle and to support photorespiration during dark-light transitions. As reported by Shikanai and Yamamoto (2017), under steady-state conditions, Flvs have poor access to its putative electron donor Fd, due to activation of the Calvin–Benson cycle, but may regain functionality under highly reduced stromal conditions. Here, *A. thaliana* plants expressing cyanobacterial *Flv1/Flv3* responded differentially to the growth light conditions (Figure 1). Under low light intensity, electron transport is typically limited by the availability of photons, so that there is no need of relief with respect to the electron pressure on the PETC. The latter becomes important as the light intensity increases, and the heterologous Flv system was able to dissipate the surplus of reducing equivalents as long as the intensity did not become excessive, as observed for the stressed phenotypes of plants grown at 600 μmol photons m^–2^ s^–1^ and showing anthocyanin accumulation (Figure 1B). When exposed to a long-day regime (16-h photoperiod), the presence of the *Flv* transgenes accelerated flowering (data not shown), and plant biomass accumulation and seed yield were boosted (Figures 2B,C), illustrating the benefits of Flv1/Flv3 as additional electron sink under these growth conditions.

In chloroplasts, ATP is generated via the linear and CET pathways (DalCorso et al., 2008), while the mitochondrial respiratory electron transport chain makes an additional contribution during both dark and daylight hours (Liang et al., 2015; Voon et al., 2018). ATP levels were higher in the leaves of *Flv*-expressing transgenics than in those of WT plants after exposure to either 4 or 8 h of light, suggesting that the Flv1/Flv3 dimer was able to dissipate electrons at PSI, enhance linear electron flow and thereby establish the pH gradient required for ATP synthesis (Figure 4a). The adenylate pool is also an important regulator of plant metabolism (Geigenberger et al., 2010). In the *Flv* transgenics, an increased ATP level served to boost the activity of the Calvin–Benson cycle, which in turn helped to maintain a high level of hexose phosphates at the middle of the light period (Figures 5A,B). Hexose phosphates and ADPGlc accumulated by *Flv*-expressing plants (Figures 5A–D) were most likely used to synthesize sucrose and starch during the day, serving to stimulate plant growth (Figures 3A,B). The lack of effect of *Flv* expression on the levels of the TCA cycle intermediates citrate and malate implies that these organic acids most likely play at best a minor role in determining biomass production (Supplementary Figures S5a–d).

Manipulation of plastid levels of adenylate kinase was shown to increase starch and amino acid contents in potato (Regierer et al., 2002), and to boost the accumulation of amino acids and promote growth in *A. thaliana* (Carrari et al., 2005). In the *Flv* transgenics exposed to 4 h of light, however, there was no evidence for any significant alteration in the leaf’s amino acid contents with the exception of alanine (lines L1, L3) and GABA (lines L2, L3), suggesting that by this time illuminated leaves converted most of their photoassimilate into starch (Supplementary Table S3). In contrast, the contents of asparagine, arginine, glycine, glutamine, alanine and proline were raised by the plant’s exposure to 8 h of light (Supplementary Table S4), consistent with the observation that an increase in carbon availability enhances the assimilation of the nitrogen needed for protein synthesis and hence for the continuation of growth in the absence of light (Lawlor, 2002). The glutathione pool was more oxidized in leaves of *Flv*-expressing plants than in their WT siblings (Supplementary Figure S4). This was an unexpected result, considering that the presence of Flv1/Flv3 should inhibit the leakage of electrons from the PETC to O_2_. It is conceivable that cellular compartments and organelles other than chloroplasts contribute the higher GSSG levels observed in the transgenics (Supplementary Figure S4b), but further research will be necessary to properly substantiate this hypothesis.

The levels of both sucrose and ATP were higher in the *Flv* transgenics than in their WT counterparts, not only during illumination, but also during the dark period. According to Sharkey et al. (2004), the levels of sucrose and ATP are highly dependent on carbon metabolism during the night, while Sulpice et al. (2009) and Graf and Smith (2011) have shown that these levels constitute important determinants of biomass accumulation. It seems therefore likely that the growth advantage enjoyed by the *Flv* transgenics reflects their superior capacity to generate photoassimilate and ATP.

**FIGURE 6:**
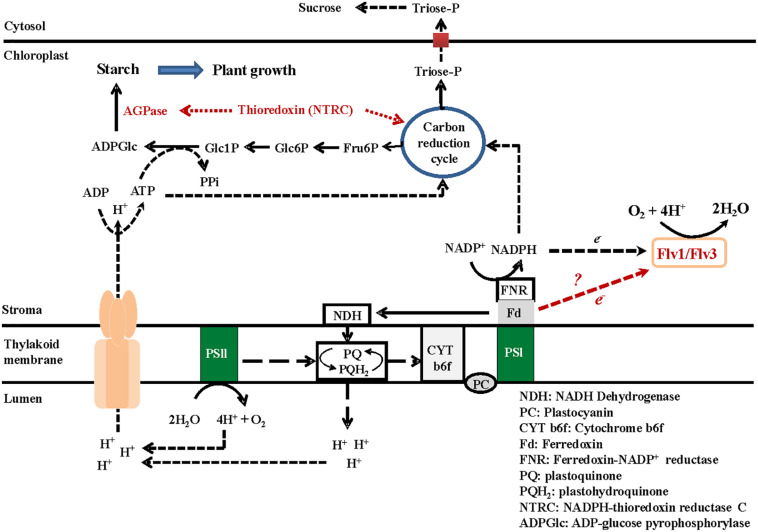
A model describing the metabolic consequences of heterologously expressing *Flv* genes in the chloroplasts of *A. thaliana*. The presence of Flv1/Flv3 creates an electron sink and balances the surplus of the electron flow through PSI and PSII by delivering these electrons to oxygen, which is converted to water. The production of redox equivalents such as NADPH is maintained, resulting in the recycling of carbon through the Calvin–Benson cycle, which in turn generates an increased supply of the phosphorylated metabolites needed for starch synthesis. The energy required for this reaction is provided by ATP, which is synthesized by the H^+^-ATP synthase driven by protons pumped by the PETC. The continuous flow of electrons results in acidification of the lumen, which is the driving force for ATP synthesis. ATP is used for the conversion of Glc1P to ADPGlc via ADPGlc pyrophosphorylase. An increased availability of ADPGlc supports a higher level of starch synthesis; starch accumulates when the leaf is exposed to light and is degraded during the dark phase.

Overall, heterologously expressing *Flv1/Flv3* in *A. thaliana* appeared to impact central metabolic routes increasing ATP levels for carbon assimilation and other biosynthetic pathways, and favoring the use of reducing equivalents in productive processes, ultimately boosting growth at moderate to high light intensities (Figure 6).

## Conclusion

The present data have demonstrated that Flv proteins contribute to the efficient functioning of the PETC and that can be introduced in angiosperms with growth and eventually yield benefits. We show that Flvs can act as additional electron sinks when expressed in *A. thaliana*, delivering any excess of reducing equivalents to oxygen, and generating the phosphorylated metabolites required for starch synthesis. The energy needed for this reaction is provided by ATP, which is produced via electron transport and lumen acidification. ATP is also used for the conversion of Glc1P to ADPGlc, mediated by ADPGlc pyrophosphorylase activity. The promotion of ADPGlc finally results in an enhanced level of starch synthesis in leaves exposed to light, and the accumulated starch is metabolized during the dark phase allowing for a continuous growth of the plant (Figure 6).

## Data Availability Statement

All datasets generated for this study are included in the article/Supplementary Material.

## Author Contributions

MH and NC made substantial contributions to conception and design, interpretation of the results, and preparation of the manuscript. ST made all practical work and did the acquisition and analysis of data, and contributed to preparation of the manuscript. FS and MM were involved in drafting and revising the manuscript, and preparation of the figures. TR, RG, and AL were involved in preparation of microscopy figures (TR), interpretation of the data, and revising the manuscript (RG and AL). NW contributed to the final revision and gave the final approval of the manuscript. All authors contributed to the article and approved the submitted version.

## Conflict of Interest

The authors declare that the research was conducted in the absence of any commercial or financial relationships that could be construed as a potential conflict of interest.

## Footnotes

1 https://imagej.nih.gov/ij

2 www.sigmaplot.co.uk/products/sigmaplot/sigmaplot-details.php

## Supplementary Material

FIGURE S1 Expression of Flv1/Flv3 in *A. thaliana.* Levels of *Flv* transcripts in the transgenic plants as determined by qRT-PCR. Experimental details are given in section “Materials and Methods.” L1–L3: independent lines harboring *Flv1/Flv3*.

FIGURE S2 Subcellular localization of recombinant Flv1/Flv3. **(a)** Schematic representation of the binary vectors for localization of the *Flv1/GFP* and *Flv3/GFP* fusion transgenes transiently expressed in *N. benthamiana*. The *Flv* genes were fused in-frame to DNA sequences encoding the pea FNR transit peptide (TP) at their 5′-end and *GFP* at their 3′-end, taking advantage of PGBW5 Gateway binary vectors driven by the CaMV 35S promoter. **(b)** GFP fluorescence in the chloroplasts of *N. benthamiana* transformed with *GFP*-tagged *Flv1* and *Flv3* genes. The left panels show GFP fluorescence, the central panels, chlorophyll autofluorescence and the right panels, the merged images.

FIGURE S3 Diurnal variation in the sugar contents of rosette leaves in 6 weeks old plants heterologously expressing *Flv1/Flv3* genes. Levels of **(a)** glucose and **(b)** fructose were determined in transgenic leaves harboring *Flv1/Flv3*. Data shown as means ± SE (*n* = 5).

FIGURE S4 The effect of heterologously expressing *Flv1/Flv3* genes on the contents of GSH **(a)** and GSSG **(b)**, and on the ratio of GSH to GSSG **(c)**. Rosette leaves were sampled at the end of the light period (8-h photoperiod). Data shown as means ± SE (*n* = 5).

FIGURE S5 The effect of heterologously expressing *Flv1/Flv3* genes on the leaf contents of organic acids. Malate **(a,c)** and citrate **(b,d)** were measured after plants had been exposed to 4 h **(a,b)** or 8 h **(c,d)** of light. L1–L3: lines harboring *Flv1/Flv3*. Data shown as means ± SE (*n* = 5). ^∗^, ^∗∗^: means differ from the performance of WT plants at *P* ≤ 0.05 and *P* ≤ 0.01, respectively.

TABLE S1 List of primers used for PCR and qRT-PCR determinations.

TABLE S2 Protocol for combined conventional and microwave-driven fixation, dehydration and resin embedding of *A. thaliana* leaf tissue for ultrastructural analysis.

TABLE S3 Amino acid contents in WT and transgenic lines expressing *Flv1/Flv3* genes. Measurements were carried out after 4 h of illumination. Plants were 6 weeks old. Results are expressed as means ± SE of 5 independent replicates. Significant differences are indicated by asterisks according to Student’s *t*-test (^∗^*P* ≤ 0.05).

TABLE S4 Amino acid contents in WT and transgenic lines expressing Flv1/Flv3 genes. Measurements were carried out after 8 h of illumination. Plants were 6 weeks old. Results are expressed as means ± SE of 5 independent replicates. Significant differences are indicated by asterisks according to Student’s *t*-test (^∗^*P* ≤ 0.05 and ^∗∗^*P* ≤ 0.01).
