# Supplementary figures and images for: Providing an Additional Electron Sink by the Introduction of Cyanobacterial Flavodiirons Enhances Growth of A. thaliana Under Various Light Intensities
Source: Front Plant Sci. 2020 Jun 25;11:902. doi: 10.3389/fpls.2020.00902 (PMC7330091; doi:10.3389/fpls.2020.00902)

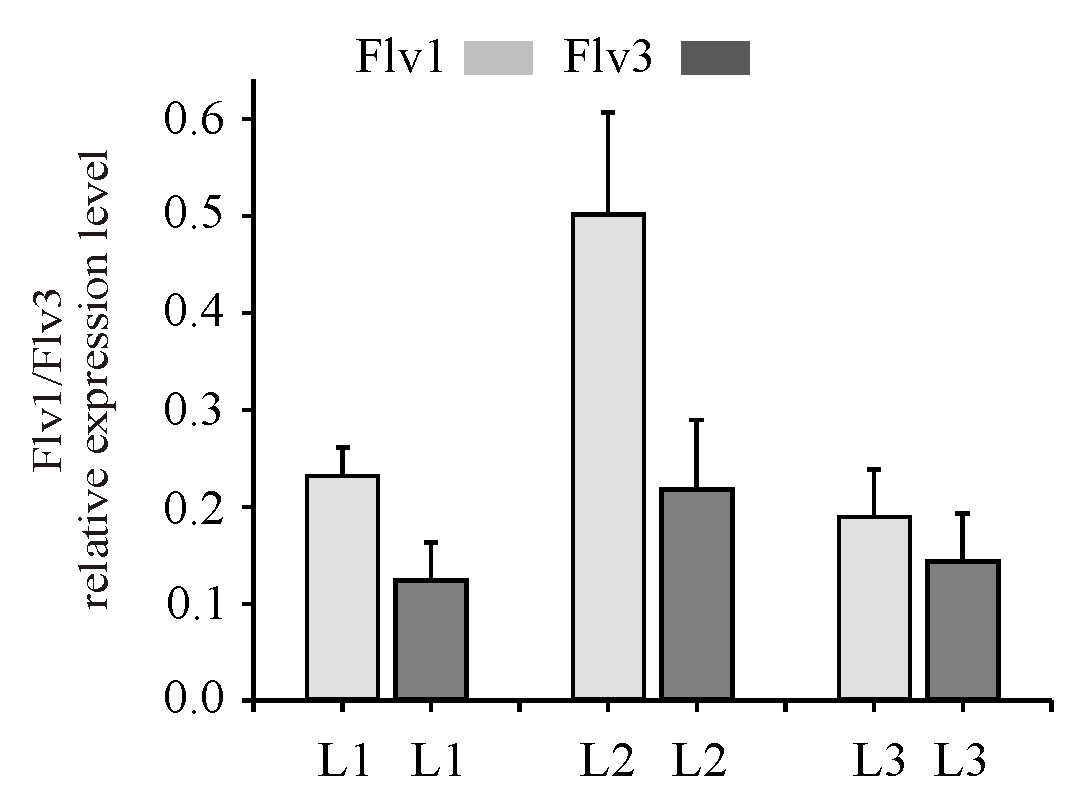

Supplement: FIGURE S1 — Expression of Flv1/Flv3 in A. thaliana. Levels of Flv transcripts in the transgenic plants as determined by qRT-PCR. Experimental details are given in section “Materials and Methods.” L1–L3: independent lines harboring Flv1/Flv3. [file Image_1.TIF]

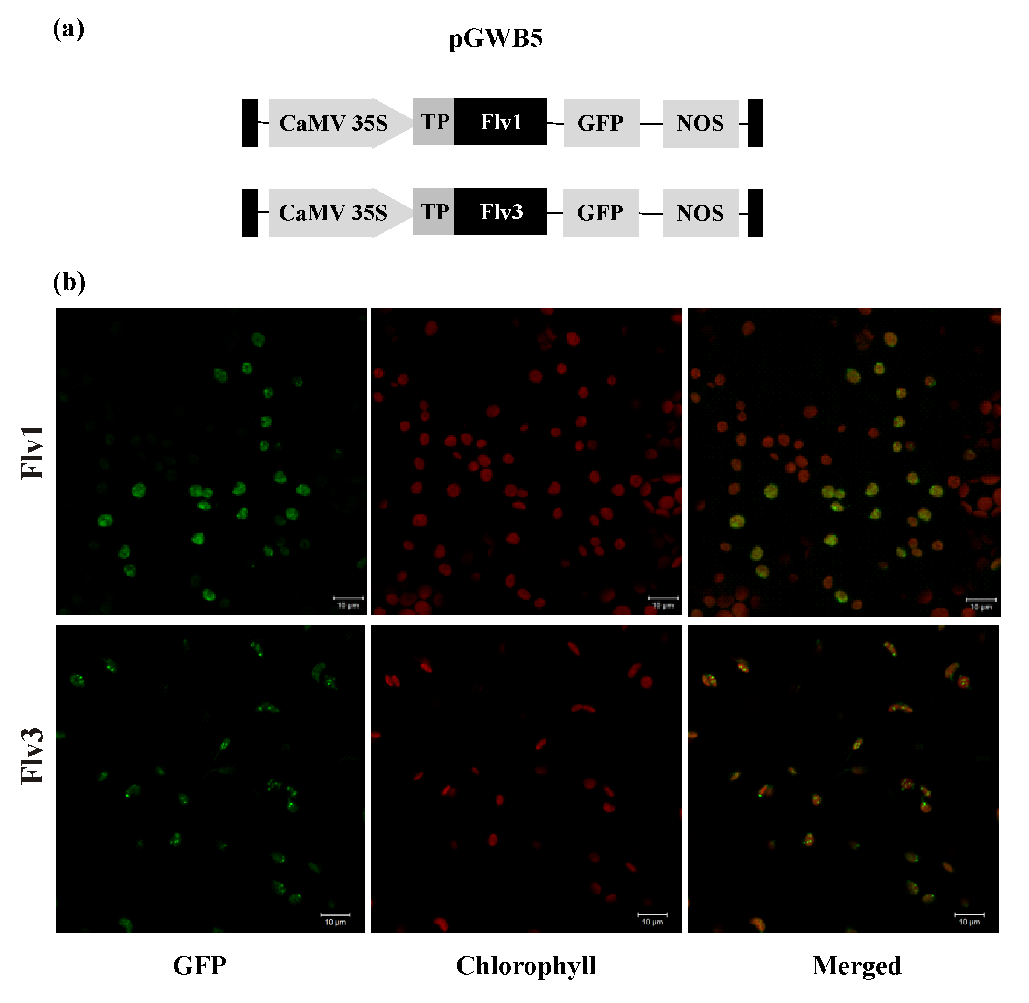

Supplement: FIGURE S2 — Subcellular localization of recombinant Flv1/Flv3. (a) Schematic representation of the binary vectors for localization of the Flv1/GFP and Flv3/GFP fusion transgenes transiently expressed in N. benthamiana. The Flv genes were fused in-frame to DNA sequences encoding the pea FNR transit peptide (TP) at their 5′-end and GFP at their 3′-end, taking advantage of PGBW5 Gateway binary vectors driven by the CaMV 35S promoter. (b) GFP fluorescence in the chloroplasts of N. benthamiana transformed with GFP-tagged Flv1 and Flv3 genes. The left panels show GFP fluorescence, the central panels, chlorophyll autofluorescence and the right panels, the merged images. [file Image_2.TIF]

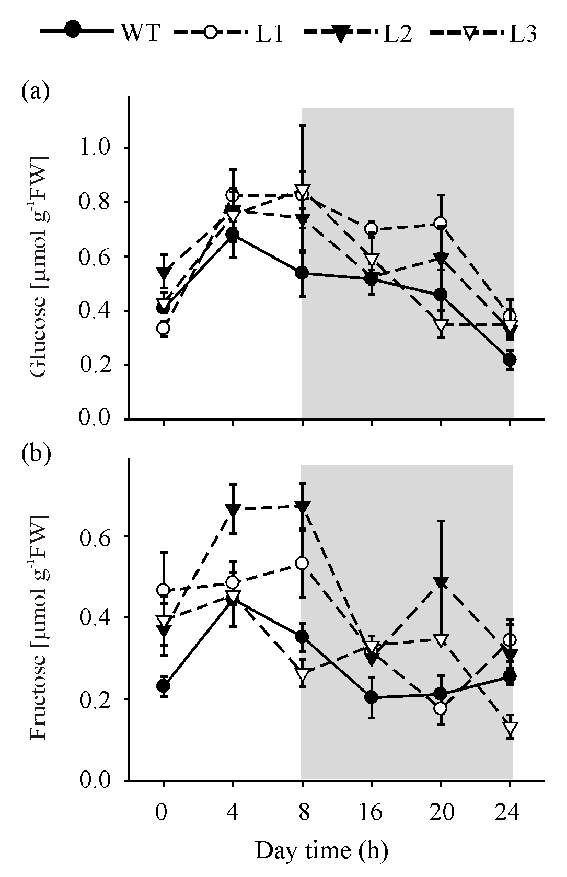

Supplement: FIGURE S3 — Diurnal variation in the sugar contents of rosette leaves in 6 weeks old plants heterologously expressing Flv1/Flv3 genes. Levels of (a) glucose and (b) fructose were determined in transgenic leaves harboring Flv1/Flv3. Data shown as means ± SE (n = 5). [file Image_3.TIF]

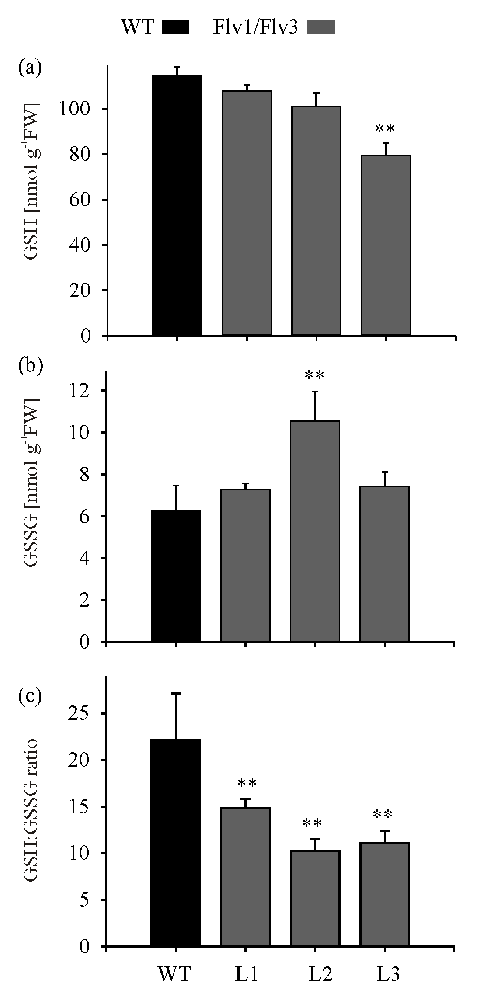

Supplement: FIGURE S4 — The effect of heterologously expressing Flv1/Flv3 genes on the contents of GSH (a) and GSSG (b), and on the ratio of GSH to GSSG (c). Rosette leaves were sampled at the end of the light period (8-h photoperiod). Data shown as means ± SE (n = 5). [file Image_4.TIF]

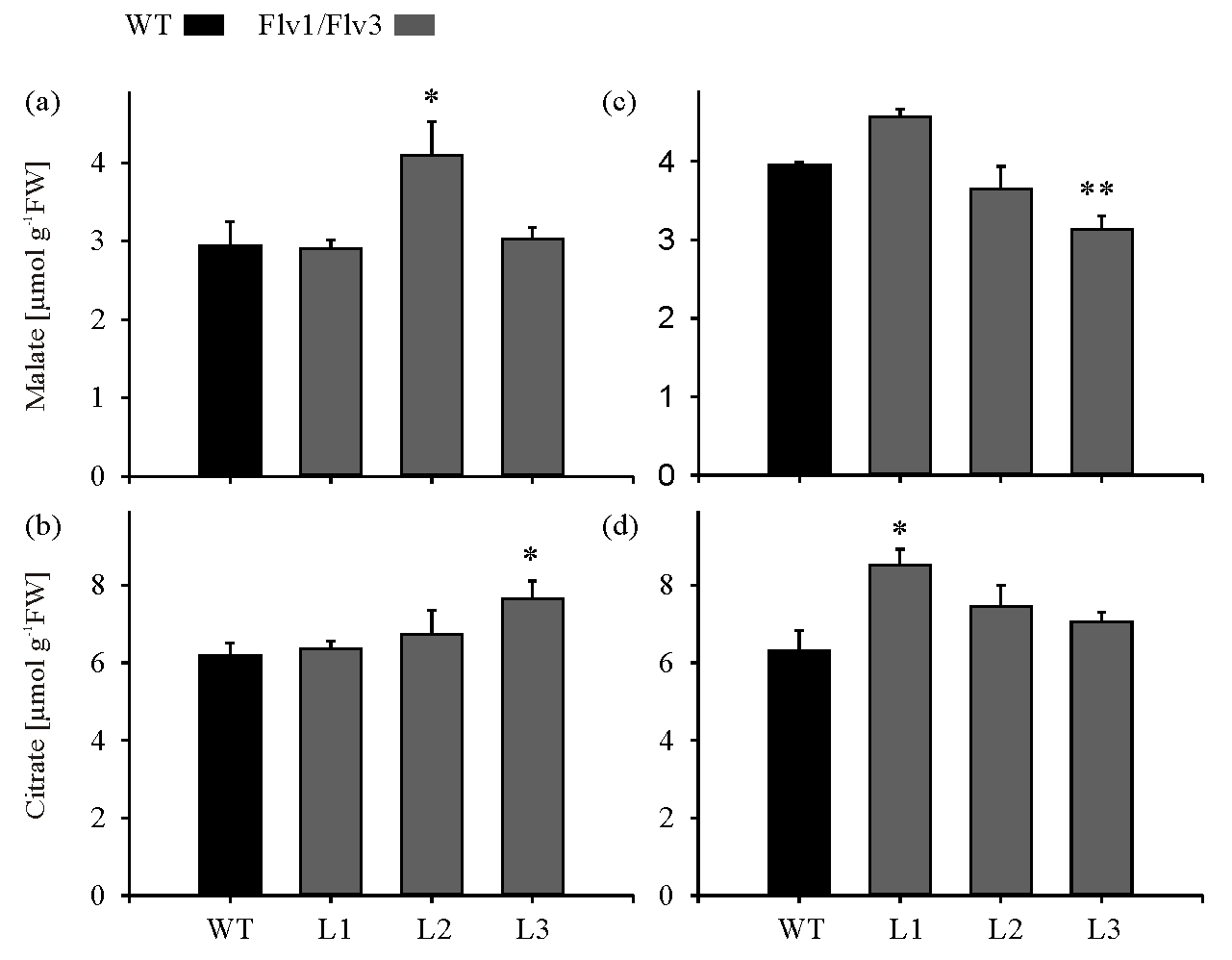

Supplement: FIGURE S5 — The effect of heterologously expressing Flv1/Flv3 genes on the leaf contents of organic acids. Malate (a,c) and citrate (b,d) were measured after plants had been exposed to 4 h (a,b) or 8 h (c,d) of light. L1–L3: lines harboring Flv1/Flv3. Data shown as means ± SE (n = 5). ∗, ∗∗: means differ from the performance of WT plants at P ≤ 0.05 and P ≤ 0.01, respectively. [file Image_5.TIF]
