# Supplementary material for: Providing an Additional Electron Sink by the Introduction of Cyanobacterial Flavodiirons Enhances Growth of A. thaliana Under Various Light Intensities
Source: Front Plant Sci. 2020 Jun 25;11:902. doi: 10.3389/fpls.2020.00902 (PMC7330091; doi:10.3389/fpls.2020.00902)
Supplement: TABLE S1 — List of primers used for PCR and qRT-PCR determinations. [file Table_1.DOCX]

**Supplementary Table S1**: List of primers used for PCR and qRT-PCR analysis

**Gene Forward primer Reverse primer**

**__________________________________________________________________________________________­_____**

***Flv1***  ATGGGAATCCATGCAAAACTGGAGAC ATAATGATCGCCAGATTTCCGGTG

***Flv3*** ATGTTCACTACCCCCCTCCCCCCCCAAAAGC GTAATAATTGCCGACTTTGCGAT

***Flv1*-RT** TGTTTGGTTCCTTCGGTTGG TTCAAAGTTTGGTCGGTGGG

***Flv3*-RT** TAAAAACCCAAACCGCAAGCA CCTTCTGCTTTAGCTACCCGA

***Ubi10*** CTTCGTCAAGACTTTGACCG CTTCTTAAGCATAACAGAGACGAG

**________________________________________________________________________________________________________**

**Supplementary Table S2:** Protocol for combined conventional and microwave-driven fixation, dehydration and resin embedding of Arabidopsis leaf tissue for ultrastructural analysis.

| **Micowave processing in a PELCO Bio Wave®Pro+(Ted Pella, Inc., Redding CA, USA)** | | | | |
| --- | --- | --- | --- | --- |
| **Process** | **Reagent** | **Power [W]** | **Time [sec]** | **Vacuum**  **[mm Hg]** |
| 1. **1. Primary fixation** | 2.0% (v/v) glutaraldehyde and  2.0% (v/v) paraformaldehyde  in 0.05 M cacodylate buffer (pH 7.3) | 150  0  150  0 | 60  60  60  60 | 5  5  5  5 |
|  |  | + overnight on shaker at 20°C | | |
| **2. Wash** | 1x 0.05 M cacodylate buffer (pH 7.3) &  2x aqua dest. | 150 | 45 | 0 |
| **3. Secondary fixation** | 1% (v/v) osmiumtetroxide in aqua dest. | 80  0  80  0 | 120  60  120  60 | 5  5  5  5 |
| **4. Wash** | 3x aqua dest. | 150 | 45 | 0 |
| **5. Dehydration** | Acetone series:30%, 40%, 50%, 60%, 70%, | 150 | 45 | 0 |
|  | 80%, 90%, 1x 100% and 1 x propylenoxide | + 5 minutes on shaker for each step | | |
| **6. Resin infiltration** | Spurr´s resin in propylenoxide on shaker : 25%, 50%, 75% for 2 hrs each, and 100% Spurr´s resin overnight on shaker | | | |
| **8. Polymerisation** | Flat embedding moulds; 24 hrs at 70°C in a heating cabinet. | | | |

**Supplementary Table S3**: Amino acid contents in WT and transgenic lines expressing *Flv1/Flv3* genes. Measurements were carried out after 4 h of illumination. Plants were six weeks old. Results are expressed as means ± SE of 5 independent replicates. Significant differences are indicated by asterisks according to student’s *t*-test (**P* ≤ 0.05).

| **Amino acids [nmol g^-1^ FW**] | **WT** |  | **Flv1/Flv3** |  |
| --- | --- | --- | --- | --- |
|  |  | **L1** | **L2** | **L3** |
| **Asparagine** | 369.8±21 | 317.8±26 | 372.18±27 | 353.2±24 |
| **Serine** | 1197±109 | 1339±52 | 1410±102 | 1427±69 |
| **Arginine** | 22.3±1.0 | 20.5±2.1 | 29.8±3.7 | 47.9±20 |
| **Glycine** | 10.8±2.3 | 13.9±1.8 | 10.8±1.3 | 7.1±1.7 |
| **Glutamine** | 903.1±95 | 842.7±48 | 947±86 | 839.5±20 |
| **Aspartate** | 752.3±65 | 722.6±58 | 803.6±100 | 740.7±14 |
| **Glutamate** | 1742±131 | 1626±117 | 1756±176 | 1718±51 |
| **Threonine** | 649.9±66 | 628.7±43 | 751.8±46 | 733.2±22 |
| **Alanine** | 628.8±50 | **478.3±23*** | 609.9±97 | **486.1±32*** |
| **GABA** | 39.2±7.5 | 55.3±8.7 | **67.2±7.9*** | **64.1±3.1*** |
| **Proline** | 576.4±106 | 646.0±67 | 600.9±11 | 603.1±81 |
| **Lysine** | 19.2±1.7 | 23.2±2.7 | 27.0±4.2 | 28.7±1.1 |
| **Valine** | 63.4±4.7 | 58.9±6.1 | 67.8±9.6 | 77.2±14 |
| **Isoleucine** | 53.6±4.8 | 57.6±5.0 | 67.7±11 | 64.8±4.8 |
| **Leucine** | 17.7±1.6 | 18.6±1.9 | 23±4.0 | 21.8±2.2 |
| **Phenylalanine** | 14.6±1.4 | 16.9±2.4 | 19.1±3.8 | 22.2±3.1 |
| **Histidine** | 4.68±1.0 | 4.9 ± 0.7 | 5.7±1.0 | 6.7±0.8 |

**Supplementary Table S4**: Amino acid contents in WT and transgenic lines expressing *Flv1/Flv3* genes. Measurements were carried out after 8 h of illumination. Plants were six weeks old. Results are expressed as means ± SE of 5 independent replicates. Significant differences are indicated by asterisks according to student’s *t*-test (**P* ≤ 0.05 and ***P* ≤ 0.01).

| **Amino acids [nmol g^-1^ FW]** | **WT** |  | **Flv1/Flv3** |  |
| --- | --- | --- | --- | --- |
|  |  | **L1** | **L2** | **L3** |
| **Asparagine** | **265±6.7** | **325±23*** | **332±19*** | **405±59*** |
| **Serine** | 991±17 | 948.4±77 | 966±13 | 945±22 |
| **Arginine** | **12.9±1.8** | **18.4±0.9*** | 14.8±0.5 | **20.2±2.0*** |
| **Glycine** | **53.2±4.8** | **86.6±13*** | **95.0±6.4**** | **120±10**** |
| **Glutamine** | **1107±36** | **1502±77**** | **1371±69*** | **1439±33**** |
| **Aspartate** | 570.9±15 | 712.8±51 | 615.9±19 | 650.1±35 |
| **Glutamate** | 1275±48 | 1194±57 | 1315±69 | 1300±40 |
| **Threonine** | 574.3±12 | 700.9±51 | 670.5±26* | 660±53 |
| **Alanine** | **437±25** | **685±32**** | **602±35**** | **551±18**** |
| **GABA** | 23.9±2.7 | 25.3±2.1 | 32.1±4.7 | 32.7±4.9 |
| **Proline** | **402±24** | **588±60*** | **656±65*** | **551±26**** |
| **Lysine** | 22.9±1.2 | 24.2±1.3 | 23.5±1.8 | 28.0±1.7 |
| **Valine** | 23.0±1.1 | 24.9±1.2 | 22.1±0.6 | 23.6±2.7 |
| **Isoleucine** | 55.1±1.0 | 68.4±4.9* | 61.0±2.7 | 60.2±5.2 |
| **Leucine** | 15.9±0.4 | 18.6±1.4 | 17.9±1.2 | 18.4±1.8 |
| **Phenylalanine** | 15.3±0.7 | 16.2±1.3 | 16.6±1.2 | 18.5±2.7 |
| **Histidine** | 3.95±0.3 | 4.7±0.8 | 3.6±0.9 | 5.3±1.3 |
